# From Gamma Coherence to Theta-Phase Synchronization: Task-Dependent Interhemispheric Integration in Boundary-Free Multiple-Object Tracking

**DOI:** 10.3390/brainsci15070722

**Published:** 2025-07-04

**Authors:** Yunfang Xu, Xiaoxiao Yang, Zhengye Si, Meiliang Liu, Zijin Li, Xinyue Yang, Zhiwen Zhao

**Affiliations:** 1 School of Artificial Intelligence, Beijing Normal University, Beijing 100875, China; xuyunfang@mail.bnu.edu.cn (Y.X.); yangxiaoxiao@mail.bnu.edu.cn (X.Y.); 202231081017@mail.bnu.edu.cn (Z.S.); liumeiliang@mail.bnu.edu.cn (M.L.); 202131081021@mail.bnu.edu.cn (Z.L.); 202321081046@mail.bnu.edu.cn (X.Y.); 2 Advanced Institute of Natural Sciences, Beijing Normal University, Zhuhai 519087, China

**Keywords:** EEG, functional connectivity, phase locking value (PLV), synchronization, multiple object tracking (MOT)

## Abstract

Background: Multiple-object tracking (MOT) is a cognitively demanding task involving sustained attention and interhemispheric integration. While previous studies have revealed that gamma-band coherence mediates interhemispheric integration in MOT tasks with visible internal boundaries, the neural mechanisms supporting integration without such boundaries remain unclear. This study investigated brain functional connectivity during a boundary-free MOT task. Methods: Thirty-eight healthy participants completed the task under four experimental conditions, defined by two load levels (two and four targets) and two movement configurations (within hemifield and between hemifield). Electroencephalography (EEG) activity was recorded in both the task and resting states. The phase locking value (PLV) and network properties were analyzed. Results: The behavioral results demonstrated greater accuracy under the two-target conditions than under the four-target conditions and significantly worse performance under the four-target between-hemifield condition. EEG analyses revealed increased theta-band PLV under the four-target between-hemifield condition, reflecting enhanced interhemispheric synchronization. The PLV difference between the four-target within-hemifield and between-hemifield conditions was positively correlated with the accuracy difference, suggesting that increased theta-band phase synchronization is associated with better task performance. Moreover, sex-related differences were observed, with males showing better performance, shorter click times, and higher theta-band PLV than females. Conclusions: Our study provides evidence that theta-band phase synchronization plays a critical role in interhemispheric integration during boundary-free MOT, extending previous findings on gamma-band coherence under visible-boundary conditions and offering new insights into the neural mechanisms of interhemispheric coordination.

## 1. Introduction

Multiple object tracking (MOT) is a fundamental cognitive function that is critical to various daily activities, including driving through traffic, playing sports [1,2], and identifying individuals in crowded environments. This complex visual task demands the sustained allocation of attention and cognitive resources to monitor multiple moving targets simultaneously [3,4,5]. During an MOT task, targets move between the visual hemifields, necessitating interhemispheric integration to maintain accurate tracking [6,7,8]. Understanding how the brain integrates information across hemispheres during MOT can provide insights into the broader mechanisms of attentional control and dynamic visual processing.

While the cerebral hemispheres exhibit specialized processing, the brain can coordinate specialized hemispheric functions through interhemispheric communication. Specifically, the human visual system processes information in a lateralized pattern, with each hemisphere being primarily responsible for the contralateral visual field [9]. This anatomical arrangement necessitates effective interhemispheric communication when tracking objects moving across the vertical meridian. Such communication is believed to be mediated by neural oscillatory synchronization between brain regions [10,11,12]. Among the neural oscillations implicated in inter-regional communication, both gamma- and theta-band rhythms have been extensively studied [13,14,15,16]. Gamma-band synchronization has been associated with local processing, such as feature binding and attentional orienting [17,18], whereas theta-band synchronization supports long-range coordination, particularly in tasks involving working memory, attentional control, and communication across distributed neural networks [14,19,20,21]. Recent research also highlights the role of theta–gamma coupling in facilitating inter-regional communication [22,23]. These findings imply that frequency-specific neural dynamics support effective communication depending on the task. Both theta and gamma rhythms are broadly implicated in attention, working memory, and information transfer [14,24,25]. In particular, theta-band activity is crucial to attentional selection and working memory operations [26,27], which are fundamental in MOT tasks.

A previous study demonstrated that interhemispheric integration imposes a cognitive cost when targets move across the entire visual field [28]. Bland et al. [29,30] explored this phenomenon by using an MOT paradigm that included visible internal boundaries dividing the visual field into distinct quadrants. Bland et al. [30] used visible horizontal and vertical internal boundaries during all trial types. Their findings showed that gamma-band coherence mediates interhemispheric integration under this condition, supporting the “communication through coherence” hypothesis [24]. It remains unclear whether similar or different oscillatory mechanisms support interhemispheric integration in boundary-free contexts.

To address this research gap, we designed a boundary-free MOT task to examine how the brain coordinates interhemispheric integration in the absence of internal visual boundaries. Building upon previous findings that gamma-band coherence mediates interhemispheric integration during MOT with explicit internal boundaries, our study aimed to investigate whether similar or distinct oscillatory mechanisms are involved under boundary-free conditions. Another difference from Bland et al.’s work is that our study recorded resting-state EEG both before and after the MOT task, allowing us to examine task-related changes in resting brain network properties. Furthermore, we explored the potential role of theta-band phase synchronization in interhemispheric integration during boundary-free MOT, building on previous studies on interhemispheric interaction [11,31] and rhythmic synchronization in cognitive functions [12,32]. We also examined sex-related differences in neural synchronization and behavioral performance, given known variations in visuospatial processing and cortical organization between sexes [33,34]. Finally, we analyzed how synchronization relates to tracking accuracy to assess its potential as a biomarker. We focused on the theta phase locking value (PLV) and brain network to characterize interhemispheric integration during cross-hemifield tracking, providing new insights into the oscillatory mechanisms in visual tracking.

## 2. Materials and Methods

### 2.1. Participants

We recruited 38 healthy individuals (19 males and 19 females; age range: 18–34 years; mean age: 23.53 ± 4.47 years), all of whom were undergraduate or graduate students at the time of the experiment. All the participants were right-handed and had normal or corrected-to-normal vision. All participants were naive to the purpose of the experiment and had no prior experience with MOT tasks. Before the experiment, each participant reviewed and signed an informed consent form. A financial incentive of USD 10 was awarded to individuals who participated in the study. This study followed the principles outlined in the Declaration of Helsinki and received approval from the Ethics Committee of Beijing Normal University (IRB Number: BNU202501080005).

### 2.2. Experimental Setup

The experiment was conducted in a quiet, well-lit room. The monitor used was 24 inches in size (with a resolution of 1920 × 1080 pixels and a refresh rate of 60 Hz). The participants were seated comfortably at a distance of 57 cm from the monitor, with their right hand resting on the mouse. They were instructed to remain still and motionless throughout the task, except during designated breaks. When completing the MOT task and during the resting-state eyes open (EO) periods, the participants were instructed to focus on the central cross and minimize eye movements and blinking.

Resting-state electroencephalography (EEG) measurements were obtained both before and after the main experiment. A 10 min resting-state measurement was recorded before the MOT task (pre: 5 min EO period and 5 min eyes closed (EC) period), and a 6 min resting-state measurement was recorded immediately after the MOT task (post: 3 min EO period and 3 min EC period). Before the main experiment, the participants completed a practice session consisting of 5 trials. The entire experiment took approximately 100 min to complete.

### 2.3. MOT Task

**Figure 1:**
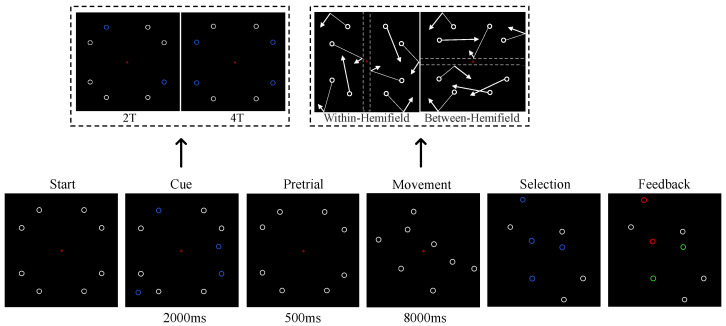
The trial procedure for the boundary-free MOT task. Circles represent the targets and distractors, while the arrows indicate their moving directions.

The design of the MOT task without internal boundaries was adapted from Bland et al.’s work [29,30]. The key difference from Bland et al. was that the internal boundaries were made invisible in our task design. All other task and trial parameters remained consistent with their study, except we did not use eye tracking. The procedural steps for each trial are illustrated in Figure 1. The participants were required to track two or four targets out of eight identical white objects. The participants were instructed to fixate on the center of the screen throughout the task. Eight white objects were evenly distributed across the four quadrants of the display at the beginning of the experiment. During the cue period, two targets (2T) or four targets (4T) turned blue to indicate which objects should be tracked by the participants. During the pretrial period, all the objects were indistinguishable (white), and the participants were required to remember the locations of the targets. During the movement period, the objects moved under either the within-hemifield or between-hemifield condition; the objects linearly deflected off all other surfaces but passed through the other moving objects. The white dashed lines represent horizontal and vertical boundaries that were not visible during the experiment. In the between-hemifield condition, the objects moved across the left and right hemifields (i.e., the objects were restricted to move within the upper and lower hemifields and were reflected when they reached the boundaries). According to the law of reflection, the angle of reflection was equal to the angle of incidence. In the within-hemifield condition, the objects were restricted to a single hemifield, and the objects could not cross between the left and right hemifields (i.e., the objects were restricted to either the left or right hemifields). During the selection period, the fixation point became a blue cursor, and the participants selected the objects they thought were the original targets using a computer mouse. Feedback (green for correct and red for incorrect) was provided after the participants completed all target selections.

The MOT task consisted of 192 trials divided into 12 blocks. Four trial types were randomly presented with equal probability within each block, including 2T-Within (2T-W), 2T-Between (2T-B), 4T-Within (4T-W), and 4T-Between (4T-B). Here, “2T” and “4T” indicate the number of targets, while “Within” and “Between” refer to within-hemifield and between-hemifield movement configurations, respectively. A break was scheduled after every 4 blocks. To minimize the potential impact of fatigue on the results, the participants determined the length of the break. Once they were ready, the participants clicked the left mouse button to continue the experiment. To improve clarity, a demonstration video of the boundary-free MOT task is provided as Supplementary Material (Video S1).

### 2.4. EEG Recording and Preprocessing

EEG signals were recorded using a portable wireless EEG system (NeuSen.W32, Neuracle, Shanghai, China), with electrodes placed according to the international 10–20 system [35]. The following electrodes were used: Fp1/2, Fz, F3/4, F7/8, FC1/2, FC5/6, Cz, C3/4, T3/4, CP1/2, CP5/6, T5/6, Pz, P3/4, PO3/4, Oz, O1/2, and A1/2 (left and right mastoids). The reference and ground electrodes were positioned at the CPz and AFz sites. The channel impedance was maintained below 15 kΩ. The signals were sampled at 1000 Hz, and offline analyses were conducted using MATLAB (R2022b).

The EEG data were preprocessed using the EEGLAB v2021.0 toolbox in MATLAB R2022b. Initially, the data of electrodes A1 and A2 were removed, and the data were re-referenced to reduce global noise. A bandpass filter with a 1–100 Hz range and a 50 Hz notch filter [36] were used to reduce power line interference. The data were then downsampled to 250 Hz to reduce computational costs. Independent component analysis (ICA) was performed using the extended infomax algorithm to decompose the data into multiple components [37,38]. The ICLable tool was used to automatically label and remove components associated with eye movements, muscle activity, line noise, and channel noise [39,40].

The EEG data were manually inspected for abnormal artifacts (e.g., jaw clenching) via a visual examination. Raw data rejection and artifact subspace reconstruction (ASR) were employed for the resting-state data to increase data quality and reliability. For the task-state EEG data, epochs were extracted for each trial, primarily focusing on the EEG signals recorded during the 8000 ms movement period. The data were subsequently analyzed across the following frequency bands: delta (δ: 2–4 Hz), theta (θ: 5–7 Hz), alpha (α: 8–12 Hz), low beta (β1: 13–25 Hz), high beta (β2: 26–35 Hz), low gamma (γ1: 36–45 Hz), mid gamma (γ2: 46–70 Hz, excluding 50 Hz), and high gamma (γ3: 71–99 Hz) [41].

### 2.5. Functional Brain Network

#### 2.5.1. Coherence

To assess functional connectivity between the cerebral hemispheres, we estimated the coherence between symmetrical pairs of EEG electrodes [30]. Coherence quantifies the degree of linear correlation between two signals in the frequency domain. Coherence values were computed using Welch’s method for spectral density estimation, in which 2 s Hamming windows with 50% overlap were employed. The coherence at a given frequency *f* was defined as

(1) $$Coherence\left(f\right)=\frac{|P_{\mathrm{xy}}{\left(f\right)|}^{2}}{P_{\mathrm{xx}}\left(f\right)P_{\mathrm{yy}}\left(f\right)}$$

where $P_{\mathrm{xy}}\left(f\right)$ is the cross-power spectral density between signals *x* and *y*, and $P_{\mathrm{xx}}\left(f\right)$ and $P_{\mathrm{yy}}\left(f\right)$ are the auto-power spectral densities of signals *x* and *y*, respectively. All power estimates are real-valued.

#### 2.5.2. PLV

The PLV quantifies the phase synchronization between two EEG signals [42,43,44], and it serves as a valuable metric for investigating the interaction and cooperation mechanisms between brain regions involved in information processing. This measure is also widely used in EEG network analysis [45]. Before PLV calculation, the EEG data were bandpass-filtered for each frequency band. The preprocessed EEG signals were first processed with the Hilbert transform to obtain the instantaneous phase of the signal. The phase difference between the two electrodes was then calculated as $\Delta \phi =\phi_{1}-\phi_{2}$, where $\phi_{1}$ and $\phi_{2}$ represent the instantaneous phases of the two electrodes. The PLV was calculated as follows:

(2) $$PLV=|\frac{1}{N}\sum_{t-1}^{N}e^{i\Delta \phi }|$$

where N is the time point. When PLV = 0, the signals between the two electrodes are not phase-locked, whereas when PLV = 1, complete phase locking occurs. The PLV was calculated via the brainstorm toolbox [46].

#### 2.5.3. Network Properties

To quantitatively assess brain functional connectivity, three commonly used network properties were calculated: clustering coefficient (Cc), global efficiency (Ge), and characteristic path length (L) [47,48,49]. Specifically, Cc reflects the degree of local interconnectedness within the network, Ge represents the efficiency of global information transfer across the network, and L measures the average shortest path length between all pairs of nodes, with smaller values indicating better overall network integration. These properties were computed based on the PLV functional networks using the Brain Connectivity Toolbox (BCT) [48,50,51].

### 2.6. Statistical Analysis

Statistical analysis was performed using GraphPad Prism version 9.0.0. To reduce the influence of extreme values on the statistical analysis, the box plot method [52] was employed for outlier detection, and the winsorization method [53] was applied for processing. The Shapiro–Wilk test [54] was employed to evaluate whether the behavioral and EEG data followed normal distributions. The normality test results, along with the corresponding Q–Q plots, are presented in Supplementary Materials, Tables S1 and S2 and Figure S1. For data following a Gaussian distribution, paired *t*-tests were conducted. If the data did not conform to a Gaussian distribution, the nonparametric Wilcoxon signed-rank test and the Mann–Whitney–Wilcoxon rank-sum test were utilized. Spearman correlation analysis was used to examine the relationships between changes (consistent increases or decreases) in PLV and accuracy in the MOT task. p<0.05 was considered to indicate statistical significance. For all multiple comparisons, the *p*-values were adjusted using false discovery rate (FDR) correction according to the Benjamini–Hochberg procedure [55].

## 3. Results

### 3.1. Behavior Performance

We calculated individual accuracy and response click time for each participant under each experimental condition (2T-W, 2T-B, 4T-W, and 4T-B). Accuracy was defined as the proportion of correctly selected targets relative to the total number of targets across all trials under each condition. Response click time was defined as the average time per target selection, calculated by dividing the total selection duration by the number of required clicks (i.e., two in the 2T conditions and four in 4T the conditions). We then computed the group mean accuracy and mean response click time by averaging across all participants for each condition.

#### 3.1.1. Accuracy in the MOT Task

**Figure 2:**
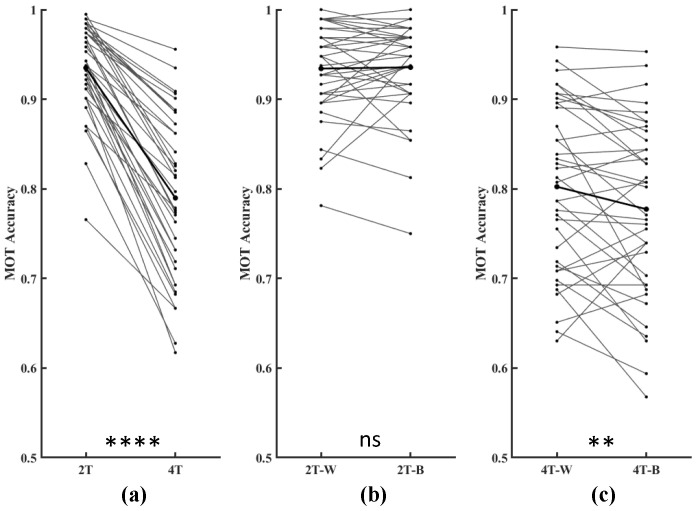
Performance in the MOT task. (**a**) Participants performed significantly better under the 2T condition than under the 4T condition. The thin lines represent the performance of individual participants. The bold black lines indicate the group means. (**b**) No significant difference in performance was observed between the 2T-W and 2T-B conditions. (**c**) Participants performed significantly better under the 4T-W condition than under the 4T-B condition, indicating a cost associated with interhemispheric integration. The statistical significance indicators are as follows: ** *p* < 0.01 and **** *p* < 0.0001; ns—not significant.

Outlier detection was systematically implemented when analyzing the behavioral data (see Supplementary Materials, Figure S2). As shown in Figure 2a, participants demonstrated significantly better performance in the 2T trials than in the 4T trials (p<0.0001). Specifically, in the 2T trials, there were no significant differences between the performance metrics under the 2T-W and 2T-B conditions (p=0.4798). However, in the 4T trials, the performance of the participants was significantly better under the 4T-W condition than under the 4T-B condition (p=0.0091).

#### 3.1.2. Sex Differences in Mean Accuracy and Mean Response Click Time in MOT Task

To examine sex-related performance differences, we analyzed the mean accuracy and mean response click time for male and female participants under each condition. The results showed distinct patterns of sex differences (Table 1). Males correctly identified significantly more targets only under the 2T-B condition, with no differences observed between male and female participants under the other conditions. In terms of click time, males responded significantly faster than females across all experimental conditions.

### 3.2. Interhemispheric Coherence and PLV Based on Paired Electrodes

**Figure 3:**
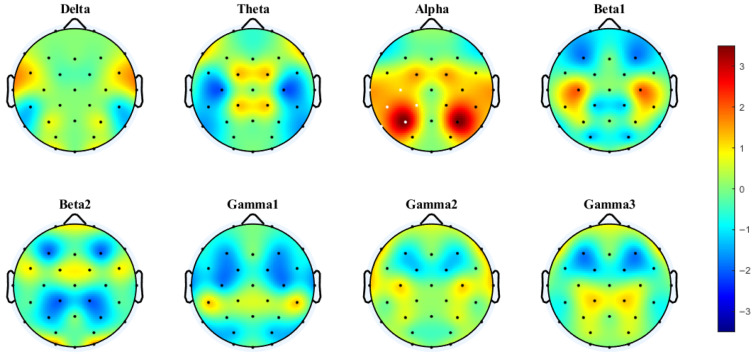
Interhemispheric coherence between the 4T-B and 4T-W conditions across eight frequency bands. The results were computed via paired *t*-tests (FDR-corrected). t-Values > 0 (in warmer colors) indicate that coherence was greater under the between-hemifield condition than under the within-hemifield condition. t-Values < 0 (in cooler colors) indicate that coherence was greater under the within-hemifield condition. Clusters were formed using a t-value threshold of |t| > 1.1526 (*p* = 0.25, df = 37) and at least two neighboring electrodes. White dots indicate electrodes included in the significant cluster identified by the cluster-based permutation test. Cluster effect sizes (Cohen’s d): alpha (0.60, *p* < 0.0001). No significant clusters were observed in other frequency bands.

**Figure 4:**
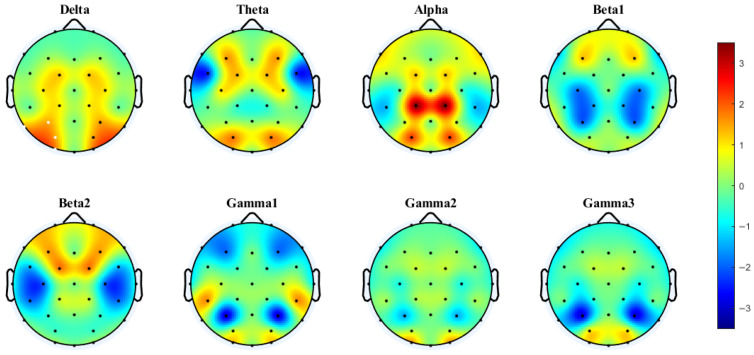
Interhemispheric coherence between the 2T-B and 2T-W conditions. White dots indicate electrodes included in the significant cluster identified by the cluster-based permutation test. Cluster effect sizes (Cohen’s d): delta (0.32, *p* = 0.0023); other frequency bands showed no significant clusters.

**Figure 5:**
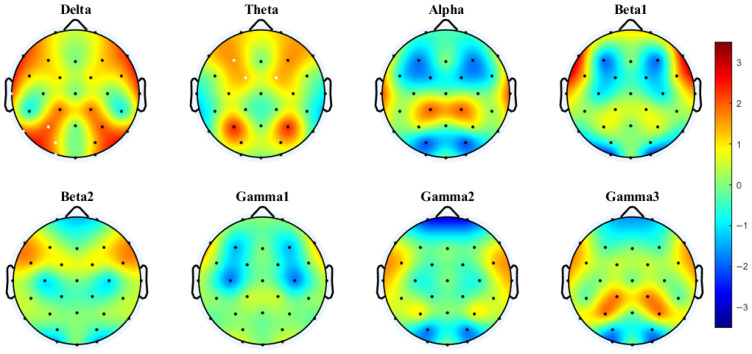
Interhemispheric PLV between the 4T-B and 4T-W conditions. Clusters were formed using a t-value threshold of |t| > 1.1526 (*p* = 0.25, df = 37) and at least two neighboring electrodes. White dots indicate electrodes included in significant clusters identified by the cluster-based permutation test. Cluster effect sizes (Cohen’s d): delta (0.46, *p* < 0.0001) and theta (0.29, *p* = 0.0013). No significant clusters were observed in other frequency bands.

**Figure 6:**
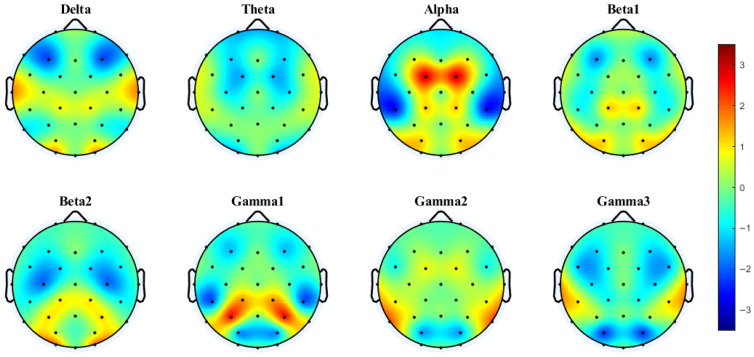
Interhemispheric PLV between the 2T-B and 2T-W conditions.

To capture the communication between cerebral hemispheres, Bland et al. [30] estimated coherence between symmetrical pairs of EEG electrodes in an MOT task with internal boundaries. We used a similar approach to investigate coherence and PLV in a boundary-free MOT task. Given the observed differences in behavioral integration costs between conditions, with significant costs evident only in the 4T trials, coherence analyses were conducted separately for the 2T and 4T conditions. The corresponding topographic maps are shown in Figure 3, Figure 4, Figure 5 and Figure 6.

Cluster-based permutation testing revealed significant differences in interhemispheric coherence between conditions. For the 4T-B vs. 4T-W comparison, only the alpha band showed significant clustering (Cohen’s d = 0.60, *p* < 0.0001), with increased coherence under the 4T-B condition occurring in the central, temporal, and parietal regions (C3–C4, CP1–CP2, CP5–CP6, P3–P4, and P7–P8). For the 2T-B vs. 2T-W comparison, significant clustering was observed in the delta band (Cohen’s d = 0.32, *p* = 0.0023), showing enhanced coherence under the 2T-B condition in the temporal and parietal–occipital regions (P3–P4, P7–P8, PO3–PO4, and O1–O2). There are no significant clusters in other frequency bands. The brain regions corresponding to the electrodes are shown in the Supplementary Materials in Figure S12.

PLV analysis demonstrated distinct patterns of interhemispheric synchronization. Under the 4T condition, significant clusters were observed in the delta (Cohen’s d = 0.46, *p* < 0.0001) and theta bands (Cohen’s d = 0.29, *p* = 0.0013). In the delta band, significant clustering was observed mainly in the temporal and parietal–occipital regions (P7–P8, P3–P4, PO3–PO4, and O1–O2). In the theta band, this enhanced synchronization under the 4T-B condition occurred in the frontal and central regions (F3–F4, FC1–FC2). No significant clustering was observed in any frequency band for the 2T comparison.

### 3.3. Network Differences

#### 3.3.1. PLV Network Analysis in Theta Band

**Figure 7:**
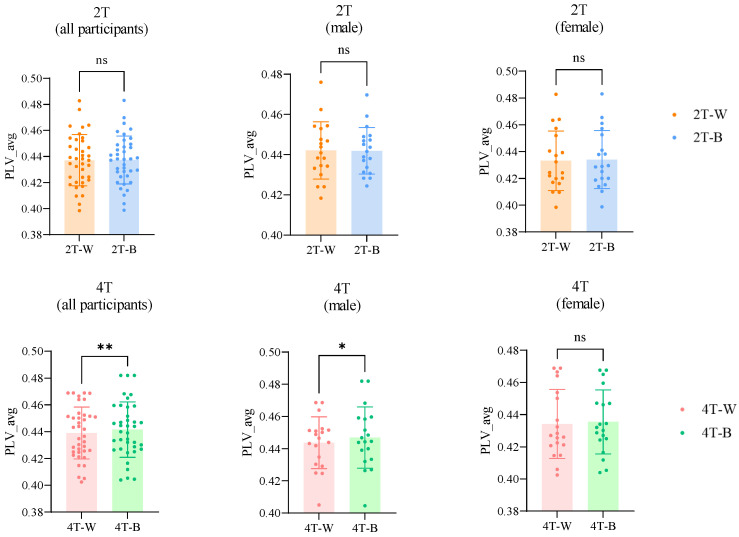
A comparison of the average PLV in the theta band across different conditions. Statistical significance is indicated as follows: * *p* < 0.05 and ** *p* < 0.01; ns—not significant.

To explore synchronization further, we conducted a whole-brain PLV network analysis across all electrode pairs. Specifically, we computed the mean PLV in the theta band to evaluate phase synchronization in brain networks. The results demonstrated significant differences in the average PLV across different conditions, as illustrated in Figure 7. No significant differences were detected between the 2T-W and 2T-B conditions for the entire participant population (*p* = 0.7663), males (*p* = 0.8894), or females (*p* = 0.2733). However, the PLV under the 4T-B condition was significantly greater than that under the 4T-W condition for all participants (*p* = 0.0089) and for males (*p* = 0.0271) but not for females (*p* = 0.3386).

#### 3.3.2. PLV Networks Across Frequency Bands

**Figure 8:**
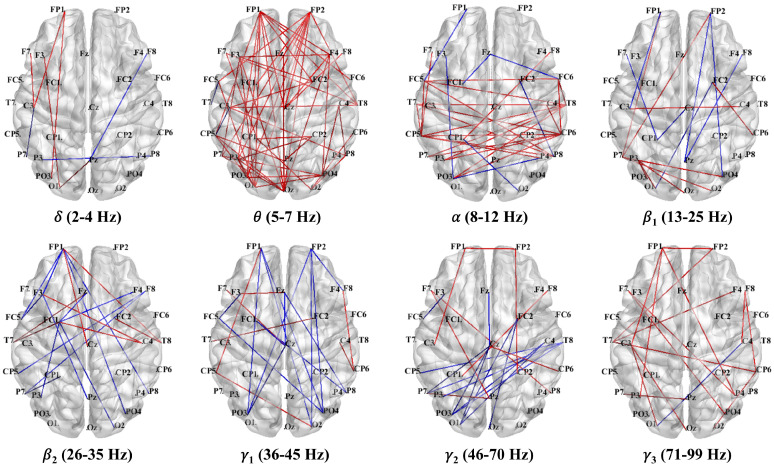
Differences in the PLV networks in eight frequency bands between the 4T-W and 4T-B conditions. The red and blue lines represent significant increases and decreases in PLV, respectively [56]. Statistical comparisons were conducted using the Wilcoxon rank-sum test with FDR correction.

As shown in Figure 8, the PLV network analyses indicated distinct synchronization patterns between the 4T-W and 4T-B conditions across frequency bands. Notably, the most prominent increase in functional connectivity under the 4T-B condition was found in the theta band, with significant interhemispheric connections. These included long-range connections between frontal and parietal–occipital regions, particularly in the left hemisphere, suggesting increased bilateral coordination with increased attentional demands.

In the delta band, the connectivity was slightly enhanced in the left hemisphere under the 4T-B condition. In the alpha band, the increased PLV indicated symmetrical cross-hemispheric connectivity without obvious lateralization. In contrast, the connectivity in the beta1, beta2, gamma1, and gamma2 bands was predominantly decreased under the 4T-B condition compared with that under the 4T-W condition, suggesting reduced synchronization in higher frequency bands in the between-hemifield tracking task. Interestingly, in the gamma3 band, the PLV was increased under the 4T-B condition, indicating the potential engagement of high-frequency activity related to cognitive processing and interhemispheric integration. Overall, a widespread increase in interhemispheric synchronization in the theta band was observed under the 4T-B condition.

**Figure 9:**
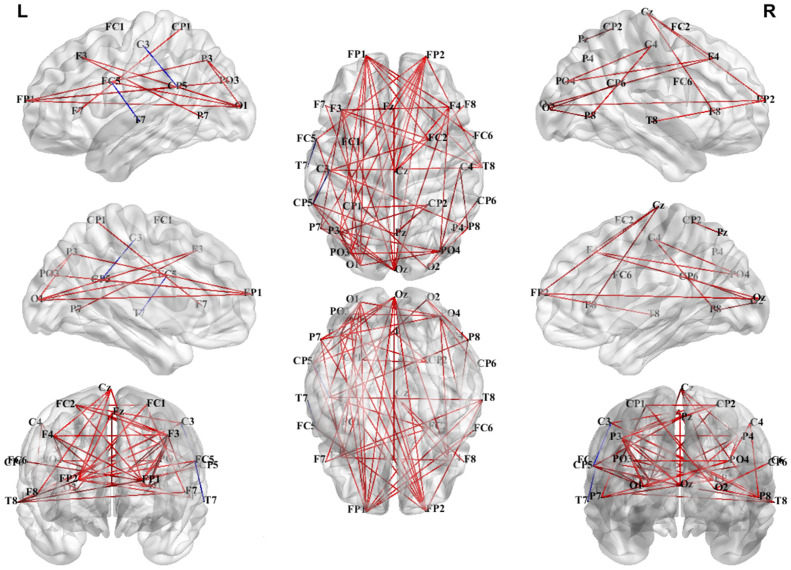
Differences in the PLV networks in the theta band under the 4T-W and 4T-B conditions.

**Figure 10:**
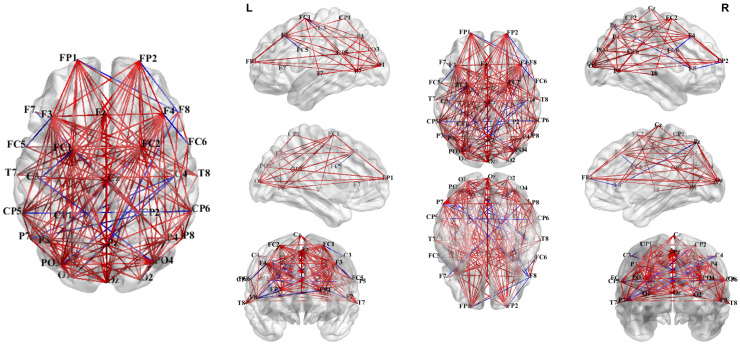
Differences in the PLV networks in the theta band under the 2T-B and 4T-B conditions.

Consistent with the average PLV results, increased connectivity in the theta band was observed in the whole-brain PLV network under the 4T-B condition compared with that under the 4T-W condition. As illustrated in Figure 9, stronger long-range functional connectivity between the parietal and occipital regions was observed in the theta-band network, with denser connections in the left hemisphere. Furthermore, as shown in Figure 10, theta-band connectivity was significantly greater under the 4T-B condition than under the 2T-B condition, indicating that the PLV increased with the number of tracked targets. The PLV networks for other frequency bands are presented in the Supplementary Materials, Figures S5–S11.

#### 3.3.3. Network Properties

**Figure 11:**
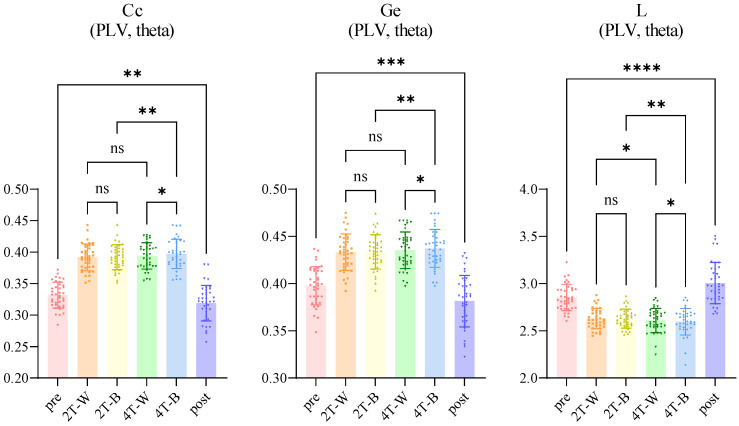
Multiple comparison results of Cc, Ge, and L in the theta band (FDR-corrected). Statistical significance was determined using Benjamini–Hochberg false discovery rate (BHFDR) correction applied across the three network properties (Cc, Ge, and L) within the theta band. Significance levels are indicated as follows: * *p* < 0.05, ** *p* < 0.01, *** *p* < 0.001, and **** *p* < 0.0001; ns—not significant.

Considering that there were significant differences in the theta-band PLV network, we analyzed theta-band PLV network properties across the pre-experiment, 2T-W, 2T-B, 4T-W, 4T-B, and post-experiment conditions. We analyzed three network properties: the clustering coefficient (Cc), global efficiency (Ge), and characteristic path length (L). The corresponding results are shown in Figure 11.

The network topology analysis revealed that task-related changes and condition-specific patterns were significant. Significant changes in all properties were observed before and after the experiment. The Cc (*p* = 0.0065) and Ge (*p* = 0.0002) values significantly decreased, whereas the L value increased (*p* < 0.0001) before and after the experiment, indicating decreased network efficiency after task completion. Condition comparisons demonstrated load-dependent network reorganization. No significant differences were found between the 2T-W and 2T-B conditions for any network properties. However, there were significant differences between the 4T-W and 4T-B conditions, with the Cc (*p* = 0.012) and Ge values (*p* = 0.0149) increasing and the L value (*p* = 0.012) decreasing under the 4T-B condition, revealing greater node clustering and shorter information transmission distances under this condition. Target load effects displayed hemisphere-specific patterns. The number of targets (two vs. four) significantly affected L under both the within-hemifield and between-hemifield conditions, with higher loads being associated with lower L. However, Cc (*p* = 0.0026) and Ge (*p* = 0.0028) were significantly modulated by the target number only under the between-hemifield condition, with no significant effects observed under the within-hemifield condition. Therefore, under the between-hemifield condition, both the clustering of network nodes and the efficiency of information transmission increased with higher target loads.

### 3.4. Correlation Between PLV and Accuracy

**Figure 12:**
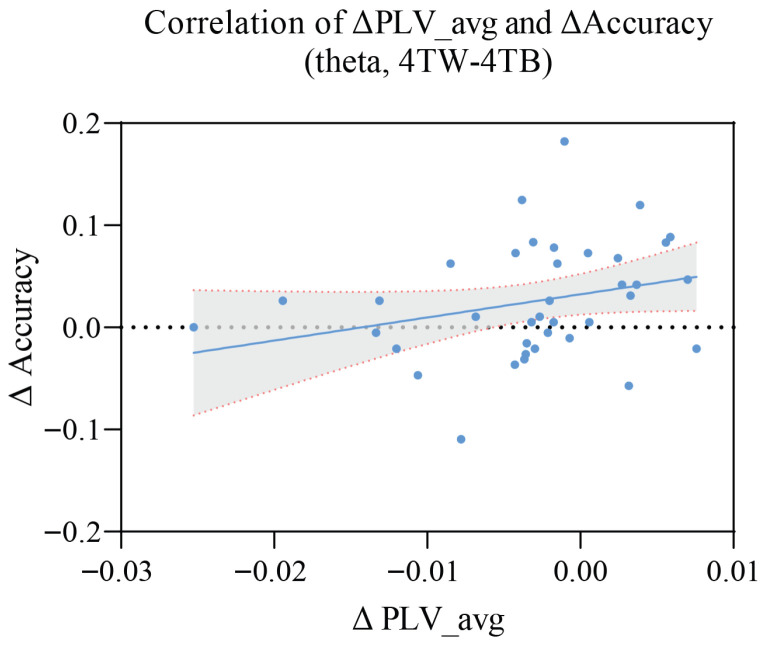
Correlation between ΔPLV_avg and ΔAccuracy under the 4T-W and 4T-B conditions.

As shown in Figure 12, we calculated the correlation between ΔPLV_avg and ΔAccuracy. ΔPLV_avg represents the mean PLV, Accuracy represents the accuracy in the MOT task, and Δ was calculated with the results of the 4T-W condition minus those of the 4T-B condition. A significant positive correlation was observed between the two measures (*p* = 0.0292), indicating that as ΔPLV_avg increased, ΔAccuracy increased. This finding implies that brain network synchronization could be used to predict task performance.

### 3.5. Analysis of Temporal Trends in Accuracy and Click Time Across Blocks

We next performed a block-wise analysis of the mean accuracy and response click time in the MOT task. We examined performance trends across 12 blocks (192 trials), indicating learning effects, fatigue effects, condition-specific differences, and sex-related differences.

**Figure 13:**
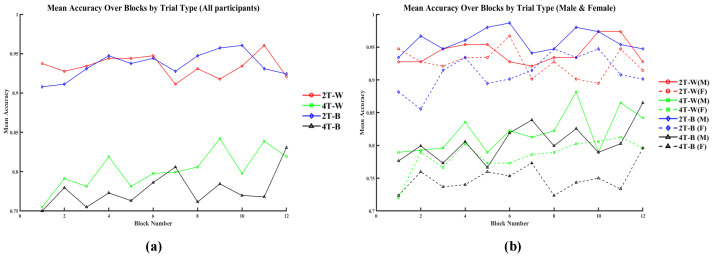
Mean accuracy for all participants (**a**) and by sex (**b**) across different task conditions.

As shown in Figure 13, across the 12 blocks, participants generally had higher accuracy in the 2T-W (red solid line) and 2T-B (blue dashed line) trials, often exceeding 0.9. In contrast, performance under the 4T-W (green solid line) and 4T-B (black solid line) conditions was lower, with increased interblock variability. The mean accuracy of all participants under the 4T conditions tended to increase over time.

**Figure 14:**
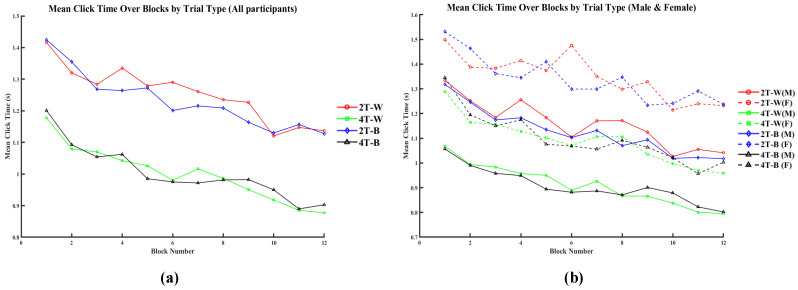
Mean click time across blocks for different trial types for all participants (**a**) and by sex (**b**).

As shown in Figure 14, the mean click time consistently decreased across all 12 blocks under all conditions. The participants exhibited longer click times in the 2T-W and 2T-B trials (red and blue solid lines, respectively) and shorter click times under the 4T-W and 4T-B conditions (green and black solid lines). Overall, the mean click times of all participants showed a downward trend, with males (solid lines) consistently demonstrating faster responses than females (dashed lines) across all trial types.

## 4. Discussion

### 4.1. Behavioral Findings in the Boundary-Free MOT Task

Consistent with previous research, behavioral performance declined as the target load increased, reflecting the capacity limitations of visual attention and working memory and providing support for the limited-capacity model of MOT [6,57]. The lack of significant differences between the within- and between-hemifield conditions in the 2T trials suggests that tracking fewer targets imposes less demand on interhemispheric integration. However, the significant performance decrease under the 4T-B condition suggests that interhemispheric integration requires additional neural resources in scenarios with higher cognitive demands [58].

We also observed sex-related differences in performance. Across all conditions, males exhibited higher accuracy than females, with significant differences under the 2T-B condition. Additionally, males exhibited significantly shorter click times than females did across all conditions. These findings are consistent with prior research indicating sex-related differences in spatial attention and motion processing [33,59,60] and imply that males may have an advantage [34,61] in certain aspects of object tracking, particularly under conditions that require interhemispheric integration. The block-wise analysis of the accuracy and response click time exhibited temporal changes in performance in MOT tasks. Accuracy increased and mean click times decreased across blocks, indicating that performance improved with task familiarity [62,63]. These learning effects were observed in both sexes. While males demonstrated significantly shorter mean click times across all conditions, a significant sex difference in accuracy was found only under the 2T-B condition, possibly reflecting sex-related differences in visuospatial processing or task strategy. The observed lack of task-dependent theta synchronization increase in females may suggest the use of alternative neural strategies for interhemispheric integration. Previous studies have shown that females often exhibit more bilateral or distributed cortical activation during spatial and cognitive tasks, which may reflect different processing styles that are less reliant on interhemispheric phase synchronization [64,65]. Additionally, behavioral differences observed between sexes in our study, such as faster response speeds and higher accuracy in males, may be related to these neural differences [66,67].

Together, these behavioral results underscore the importance of both task demands and individual differences in MOT performance. In particular, they suggest that interhemispheric integration is dynamically modulated by cognitive load and may be influenced by sex-specific neural strategies.

### 4.2. Frequency-Dependent Neural Mechanisms: Gamma Coherence Versus Theta-Band Phase Synchronization

Previous research has examined interhemispheric coherence between symmetrical pairs of EEG electrodes during MOT tasks with internal boundaries, revealing that gamma coherence was greater under the between-hemifield condition than under the within-hemifield condition [30]. While Bland et al. (2020) [30] emphasized gamma-band coherence in their interpretation, they also reported integration-related changes in the delta and theta bands, suggesting that interhemispheric integration processes may involve multiple frequency ranges.

Significant interhemispheric coherence was observed only in the delta band during the 2T trials and in the alpha band during the 4T trials. The absence of gamma-band coherence enhancement in our boundary-free MOT task may be due to the reduced spatial constraints in our paradigm. The load-dependent shift from delta-band coherence under the 2T condition to alpha-band coherence under the 4T condition may suggest that different frequencies mediate interhemispheric integration as attentional demands increase. This frequency-selective recruitment may reflect the brain’s adaptive use of different oscillatory mechanisms depending on the task context.

The delta-band synchronization enhancement observed in both coherence (2T-B) and PLV (4T-B) analyses suggests that low-frequency oscillations may play a key role in interhemispheric integration. The theta PLV increase under the 4T-B condition indicates that theta-band synchronization becomes critical as attentional demands increase. The absence of a significant PLV increase under the 2T condition may reflect the relatively lower attentional demands, requiring less interhemispheric integration. Overall, these findings demonstrate that interhemispheric connectivity is dynamically mediated in a frequency- and task-specific manner, supporting the idea of flexible cross-hemispheric integration during complex visual attention tasks.

To further explore interhemispheric integration, we constructed a whole-brain PLV network and found that the theta-band PLV was significantly higher under the between-hemifield condition than under the within-hemifield condition. This shift from gamma-band coherence to theta-band phase synchronization indicates a task-dependent mechanism underlying interhemispheric integration during the MOT task, reflecting the brain’s flexible use of oscillatory dynamics depending on the task context [68,69]. Moreover, previous studies have demonstrated that gamma oscillations are primarily involved in local feature binding, whereas theta oscillations are strongly associated with global perception and feature processing [70,71,72]. Furthermore, theta oscillations have been consistently implicated in cognitive control and working memory processes, both of which are essential to dynamic object tracking [58,73].

Bland et al. [30] used boundaries to divide the visual field into multiple regions, which allowed the brain to focus more on local features, leading to significant gamma coherence under the between-hemifield condition compared with that under the within-hemifield condition. In our boundary-free MOT task, since no boundaries were present to influence the results, the brain focused more on global perception, leading to significant PLV synchronization in the theta band. In addition, Canolty et al. [74] revealed that high-frequency brain activity reflects local domains of cortical processing, whereas low-frequency brain rhythms are dynamically entrained across distributed brain regions according to both external sensory inputs and internal cognitive events. In the MOT task with explicit internal boundaries, interhemispheric integration is facilitated by high-frequency gamma-band synchronization. Conversely, owing to the lack of explicit spatial boundaries in the boundary-free MOT task, the brain must rely more on internal cognitive processes. Bland et al. [30] used visible boundaries to divide the visual field into multiple regions, which may have encouraged the brain to focus more on local spatial features, possibly leading to enhanced gamma-band coherence under the between-hemifield condition compared with the within-hemifield condition. In our boundary-free MOT task, the absence of such visual boundaries may have shifted neural processing towards a more global perceptual strategy, as reflected by increased theta-band phase synchronization. This interpretation is further supported by the notion proposed by Canolty et al. [74], who suggested that high-frequency brain activity tends to reflect local cortical processing, whereas low-frequency rhythms are more likely to engage distributed networks in response to both external sensory inputs and internal cognitive processes. Thus, the lack of explicit spatial boundaries in our task may have led the brain to rely more on internal cognitive mechanisms to achieve interhemispheric integration. Under these conditions, low-frequency theta-band synchronization becomes more prominent, which is consistent with its established role in coordinating distributed attention networks [75]. This oscillatory shift may reflect a fundamental neural adaptation strategy: when the demarcation structure in the visual environment is removed, the brain compensates by increasing internal coordination via the synchronization of low-frequency rhythms [76].

### 4.3. Functional Brain Network Dynamics

Our PLV network analyses revealed distinctive neural synchronization patterns between the 4T-W and 4T-B conditions across eight frequency bands, providing novel insights into the neurophysiological mechanisms underlying distributed cortical networks [77,78]. Notably, we found a significant increase in theta-band connectivity under the 4T-B condition compared with that under the 4T-W condition (Figure 8 and Figure 9), likely reflecting the increased demand for interhemispheric integration. This increased theta-band synchronization manifested primarily as long-range connections between frontal and parietal–occipital regions, with particularly dense connectivity in the left hemisphere. These frontoparietal connections may reflect the top-down control of attentional processes mediated by the dorsal frontoparietal system [79]. These findings align with previous research implicating theta oscillations in the long-range neural communication necessary for attentional control [80,81] and support the critical role of neural synchronization in facilitating information transfer across hemispheres.

Moreover, the pronounced theta-band PLV increases in the left hemisphere may reflect a left-lateralized neural synchronization pattern, suggesting the engagement of left-hemispheric executive control mechanisms during interhemispheric integration. Although classical models and structural imaging studies [82] have demonstrated the right hemisphere’s specialization for visuospatial attention in most humans, recent findings indicate that hemispheric dominance for attentional processes may vary depending on task demands. For instance, ERP evidence has shown that object-based visual attention, particularly when directed toward non-spatial features such as shape, elicits stronger responses over left occipito-temporal regions, implicating a left-hemispheric advantage in selective attention and object recognition [83]. In our task, participants had to simultaneously track multiple objects as distinct entities while maintaining their individual identities across dynamic spatial trajectories. This dual demand—individuating objects while categorizing them as targets or distractors—likely recruits left-hemispheric networks specialized in object-based attention and categorical processing. Unlike purely spatial attention tasks, which typically show right-hemispheric dominance, MOT requires maintaining discrete object files for each target, treating them as bounded units rather than processing global spatial configurations. Thus, the left hemisphere’s role in feature-based and object-based attention may explain the observed lateralized theta-band synchronization, as the task prioritizes object tracking over holistic spatial processing strategies. This functional asymmetry supports the notion of a dynamic and context-dependent hemispheric specialization that adapts flexibly to the demands of bilateral attentional integration. Additionally, the observed left-lateralized pattern in the present study may be influenced by the right-handedness of all participants, suggesting that individual differences in hemispheric dominance could contribute to the effects and warrant further investigation in future studies.

In the alpha band, we observed a symmetrical increase in PLV in both the left and right hemispheres, primarily involving the central, parieto–occipital, and temporal regions. This bilateral increase may reflect a large-scale regulatory mechanism for suppressing distracting information distributed across the entire visual field. In contrast to the alpha asymmetry reported by Worden et al. [84] in a unilateral spatial attention task, attentional resources must be allocated across both hemifields in the boundary-free MOT task under the 4T-B condition, thereby potentially engaging more widespread inhibitory control [85,86,87].

Furthermore, distinct PLV modulation patterns were observed in the gamma frequency band. Specifically, under the 4T-B condition, the PLV was significantly decreased in the gamma1 and gamma2 bands, particularly for interhemispheric connections. These decreases may indicate weakened local feature binding or sensory integration processes, which are often mediated by gamma oscillations [88,89]. In contrast, an increased PLV was found in the gamma3 band under the 4T-B condition, suggesting functional dissociation between different gamma subbands. High gamma activity has been implicated in higher-order cognitive functions [90]. In addition, evidence supports that different behavioral tasks evoke distinct theta/high-gamma coupling patterns across the cortex, which may support large-scale network coordination during cognitive processing [91]. The observed increase in the PLV in the gamma3 band under the 4T-B condition may reflect theta/high-gamma coupling, facilitating the integration of distributed information under demanding attentional loads.

Moreover, the significantly denser connectivity and stronger theta-band synchronization observed under the 4T-B condition than under the 2T-B condition demonstrate the impact of the cognitive load on dynamic brain network organization. This finding suggests that the visual system recruits more extensive neural networks as the tracking load increases, which is consistent with the resource theory of MOT [58,60]. The load-dependent enhancement may reflect the recruitment of additional attentional resources to maintain performance, in line with previous EEG and magnetoencephalography (MEG) findings [92,93].

### 4.4. Network Properties Analyses

The results of network properties exhibited specific changes in brain network organization, including increased Cc and Ge values, as well as decreased L values, in the theta band under the 4T-B condition compared with those under the 4T-W condition. These changes indicate increased small-world network properties, which are associated with optimal information processing [47]. Such adaptive reorganization suggests that the brain engages more efficient network topologies when interhemispheric communication is required under high attentional load, consistent with findings from visual working memory studies [94]. Furthermore, while the target number (two vs. four) significantly affected L under both the within-hemifield and between-hemifield conditions, the Cc and Ge values were significantly modulated by the target number only under the between-hemifield condition. In addition, the post-experimental resting-state results showed decreased network efficiency, which may reflect the impact of fatigue accumulated over the experiment [95,96].

### 4.5. Correlation Between Neural Synchronization and Behavioral Performance

A positive correlation between theta-band PLV changes (4T-B vs. 4T-W) and accuracy changes further supports the functional significance of theta synchronization for task performance. Specifically, greater interhemispheric synchronization was associated with better tracking performance under high-load conditions. These findings suggest that theta-band phase synchronization can serve as a biomarker for evaluating cognitive performance in visuospatial tasks. This brain–behavior relationship is consistent with the neural efficiency hypothesis [97], which posits that more effective neural processing is associated with better task performance. The observed positive correlation between changes in PLV_avg and tracking accuracy suggests that increased theta-band synchronization facilitates interhemispheric integration during boundary-free MOT, particularly under the more demanding between-hemifield condition. This pattern conceptually parallels the findings of Bland et al.’s (2020) study [30], where a failure to increase gamma-band coherence during between-hemifield tracking was associated with poorer behavioral performance under visible boundary conditions. Together, these results support the broader notion that frequency-specific synchronization mechanisms are critical to overcoming interhemispheric processing demands. In the present study, participants who could maintain greater theta-band synchronization could more efficiently manage the additional processing demands associated with between-hemifield tracking, resulting in superior behavioral performance. This pattern parallels the findings of Bland et al.’s (2020) study [30], where both theta and gamma oscillations were task-related but only gamma coherence predicted behavioral performance. Together, these findings suggest that the oscillatory mechanisms linking neural synchronization to behavioral performance may differ depending on task demands and environmental context, such as the presence or absence of internal visual boundaries.

### 4.6. Limitations and Future Directions

In future studies, eye-tracking measures could help confirm adherence to fixation targets and be used to characterize attentional allocation strategies. It should be noted that the male and female groups were not matched for demographic or experiential variables (e.g., age, athleticism, video gaming experience, etc.). Therefore, the observed sex differences may be confounded by these unmeasured factors, and the results should be interpreted with caution. Future studies should control for these variables to accurately identify sex-related effects. Furthermore, the present study did not examine age-related influences on performance due to the limited sample size [98,99]. Therefore, we plan to recruit more participants with age stratification to investigate the potential impact of age on MOT performance systematically. Since the PLV-based functional connectivity networks in this study are undirected, future research could employ causal inference methods to construct directed brain networks that can better discover causal relationships [100]. Finally, incorporating additional neural markers, such as cross-frequency coupling [101], could provide further insights into how different oscillatory mechanisms interact to support distributed visual attention under varying cognitive demands.

## 5. Conclusions

We designed a boundary-free MOT experiment to investigate the neural mechanisms underlying interhemispheric integration during dynamic object tracking. The results showed that the synchronization pattern shifted from gamma coherence (reported in previous boundary MOT studies) to theta-band phase synchronization in our boundary-free paradigm. Behaviorally, performance decreased as the target load increased. Notably, accuracy decreased significantly under the 4T-B condition, suggesting increased interhemispheric integration under higher cognitive load. In terms of sex differences, block-wise analysis revealed that males generally showed higher accuracy and faster response click times. EEG analyses demonstrated enhanced theta-band PLV under the 4T-B condition, mainly through long-range connectivity between frontal and parietal–occipital regions with a left-lateralized pattern. Network analyses demonstrated increased Cc and Ge with decreased L in the theta band during the 4T-B trials, indicating enhanced small-world network properties for optimal information processing. The positive correlation between PLV and accuracy confirms the functional significance of theta phase synchronization under the boundary-free MOT task. These findings highlight the brain’s adaptive oscillatory mechanisms based on task, suggesting that without explicit spatial boundaries, the brain enhances internal coordination through low-frequency rhythmic synchronization to facilitate interhemispheric integration. This study provides novel insights into the neural mechanisms underlying interhemispheric integration during boundary-free MOT, highlighting the critical role of theta-band phase synchronization.

## Figures and Tables

**Table 1:** Mean accuracy and mean click time by sex under the four conditions. FDR correction was applied separately for the four accuracy comparisons and the four click time comparisons.

Condition	Males	Females	FDR-Corrected *p*-Values
Mean accuracy			
2T-W	0.9419	0.9265	0.3114
4T-W	0.8199	0.7848	0.2452
2T-B	0.9600	0.9112	0.0092
4T-B	0.8051	0.7495	0.1575
Mean click time (seconds)			
2T-W	1.1587	1.3499	0.0017
4T-W	0.9112	1.0903	0.0010
2T-B	1.1261	1.3384	0.0010
4T-B	0.9077	1.1002	0.0010

Significant difference between males and females (Mann–Whitney U test, *p* < 0.05).

## Data Availability

The raw data supporting the conclusions of this article will be made available by the authors, without undue reservation.

## Supplementary Materials

The following supporting information can be downloaded at: https://www.mdpi.com/article/10.3390/brainsci15070722/s1, Table S1: Results of Shapiro-Wilk tests for MOT performance across conditions and participant groups; Table S2: Results of Shapiro-Wilk tests for average PLV across conditions and participant groups; Figure S1: Q-Q plots of the MOT performance; Figure S2: Outlier detection for MOT performance; Figure S3: Topographical distributions of power *t*-test statistics (4T-B vs. 4T-W) across eight frequency bands; Figure S4: Topographical distributions of power *t*-test statistics (2T-B vs. 2T-W) across eight frequency bands; Figure S5: Differences in the brain functional networks in the delta band in the 4T-W and 4T-B conditions; Figure S6: Differences in the brain functional networks in the alpha band in the 4T-W and 4T-B conditions; Figure S7: Differences in the brain functional networks in the beta1 band in the 4T-W and 4T-B conditions; Figure S8: Differences in the brain functional networks in the beta2 band in the 4T-W and 4T-B conditions; Figure S9: Differences in the brain functional networks in the gamma1 band in the 4T-W and 4T-B conditions; Figure S10: Differences in the brain functional networks in the gamma2 band in the 4T-W and 4T-B conditions; Figure S11: Differences in the brain functional networks in the gamma3 band in the 4T-W and 4T-B conditions; Figure S12: Brain topography and regions covered by electrodes; Video S1: Demonstration video of the boundary-free MOT task.

## Abbreviations

The following abbreviations are used in this manuscript:

MOT	Multiple-object tracking
EEG	Electroencephalogram
PLV	Phase locking value
EO	Eyes open
EC	Eyes closed
2T-W	Two-target within-hemifield condition
2T-B	Two-target between-hemifield condition
4T-W	Four-target within-hemifield condition
4T-B	Four-target between-hemifield condition
