# Supplementary material for: From Gamma Coherence to Theta-Phase Synchronization: Task-Dependent Interhemispheric Integration in Boundary-Free Multiple-Object Tracking
Source: Brain Sci. 2025 Jul 4;15(7):722. doi: 10.3390/brainsci15070722 (PMC12293150; doi:10.3390/brainsci15070722)
Supplement: Supplementary file 1 [file brainsci-15-00722-s001.zip › brainsci-3732277-supplementary.pdf]

Supplementary Materials

1 Detailed experimental setup

Participants were seated 57 cm from the display monitor. The MOT arena had a width and height of 24° visual angle (DVA). The fixation cross consisted of four red squares and one gray square (0.2 DVA square width). Circular white objects had a diameter of 1 DVA with a line width of 0.2 DVA. Object movement speed was set to 10 DVA per second. Initial heading directions were configured to ensure objects always moved between adjacent quadrants (e.g., eliminating purely vertical movement in between-hemifield trials). Specifically, in between-hemifield trials, initial headings were uniformly sampled from ±[30–45] and ±[135–150]; in within-hemifield trials, initial headings were uniformly sampled from ±[45–60] and ±[120–135]. To prevent participants from predicting trial types, target initialization followed specific patterns: two-target trials were initialized in diagonally opposite quadrants, while four-target trials were initialized with one target in each quadrant. All target initial positions remained constant across trials, with targets starting equidistant from the fixation point. Trajectories were determined to ensure no target overlap occurred at the end of the tracking period.

2 Normality tests results

Tables S1–S2 show the Shapiro–Wilk test results for normality for all experimental conditions, separated by participant group (all participants, only males, and only females).

Table S1: Results of Shapiro-Wilk tests for MOT performance across conditions and participant groups.

Condition	Shapiro-Wilk test (p value)	Number of values
	**All participants**	
2T–W	0.0068	38
2T–B	0.0001	38
4T–W	0.0854	38
4T–B	0.3483	38
2T	<0.0001	76
4T	0.0622	76
	**Male**	
2T–W	0.0185	19
2T–B	0.0418	19
4T–W	0.7455	19
4T–B	0.9730	19
2T	0.0002	38
4T	0.5032	38
	**Female**	
2T–W	0.6773	19
2T–B	0.0956	19
4T–W	0.0277	19
4T–B	0.1093	19
2T	0.1057	38
4T	0.0068	38

Table S2: Results of Shapiro-Wilk tests for average PLV across conditions and participant groups.

**Condition**	**Shapiro-Wilk test (p value)**	**Number of values**
	**All participants**	
2T–W	0.9696	38
2T–B	0.9998	38
4T–W	0.1195	38
4T–B	0.4082	38
	**Male**	
2T–W	0.8839	19
2T–B	0.6677	19
4T–W	0.4033	19
4T–B	0.7640	19
2T	0.6179	38
4T	0.2218	38
	**Female**	
2T–W	0.3632	19
2T–B	0.5036	19
4T–W	0.0656	19
4T–B	0.3257	19
2T	0.0619	38
4T	0.0180	38

3 Q–Q plots

The Q–Q plots corresponding to the Shapiro–Wilk test results are shown in Figure S1.

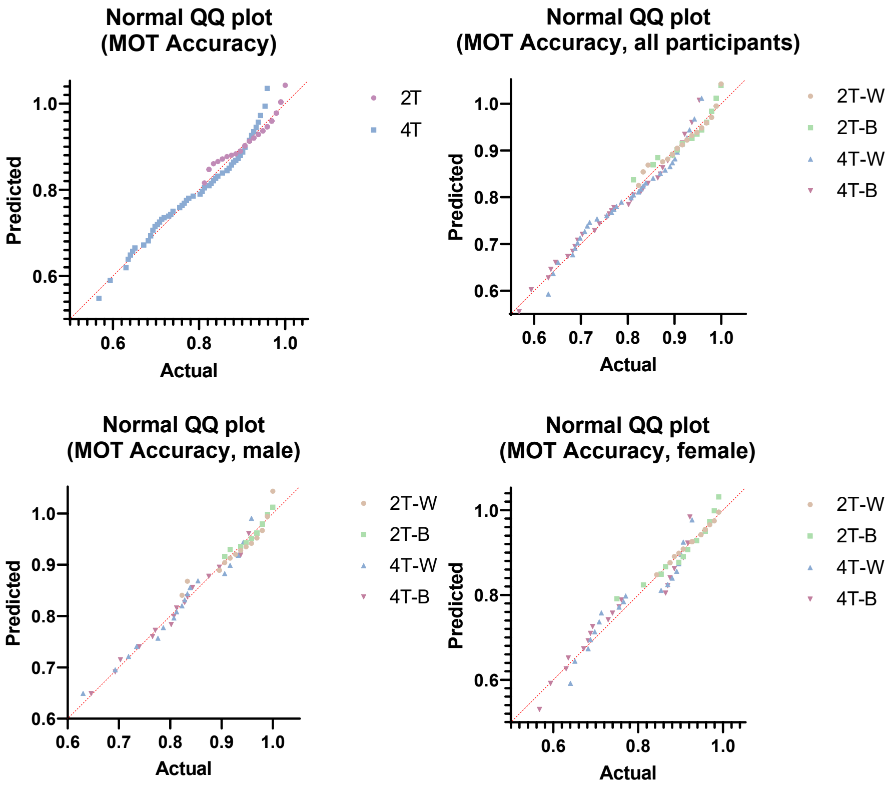

Figure S1: Q-Q plots of the MOT performance.

4 Outlier detection for MOT performance

We used the box plot method to detect outliers in the data and employed the winsorization method to replace outliers with the largest or the second smallest value in the data, as shown in Figure S2.

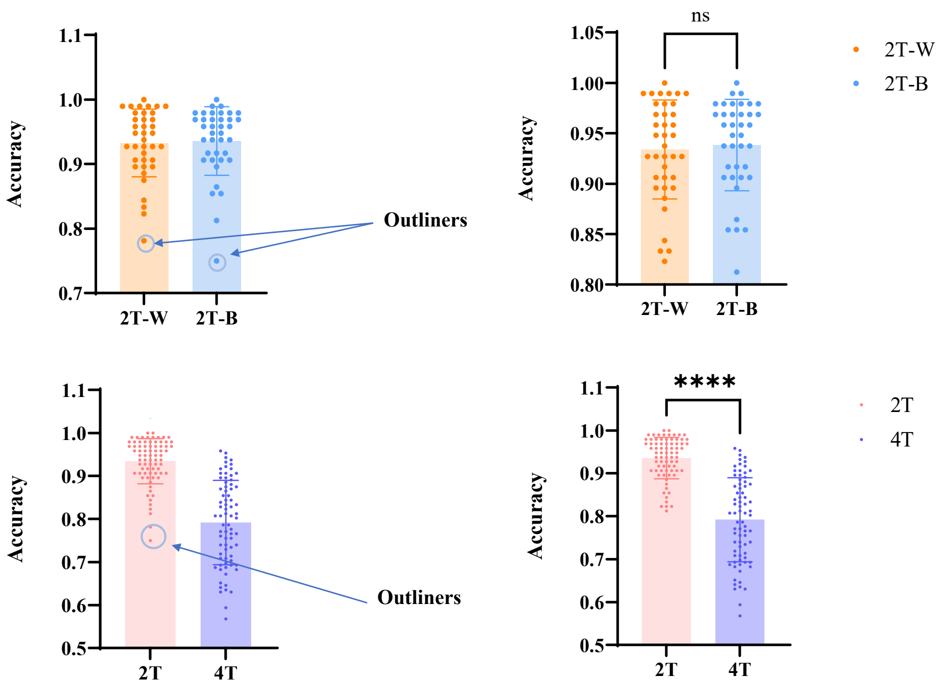

Figure S2: Outlier detection for MOT performance. The left panels show the original data with outliers, and the right panels show the results after the outliers were replaced. Statistical significance is indicated as $^{****}p < 0.0001$.

5 Topographical Distribution of Power Differences Between Between- and Within-Hemifield Conditions

To explore whether differences in interhemispheric coherence and PLV were accompanied by changes in power distribution, we conducted a power spectral analysis across eight frequency bands. Topographical distributions of t-test statistics were generated for the Between- vs. Within-Hemifield comparisons under the 4T, 2T, and combined (2T+4T) conditions.

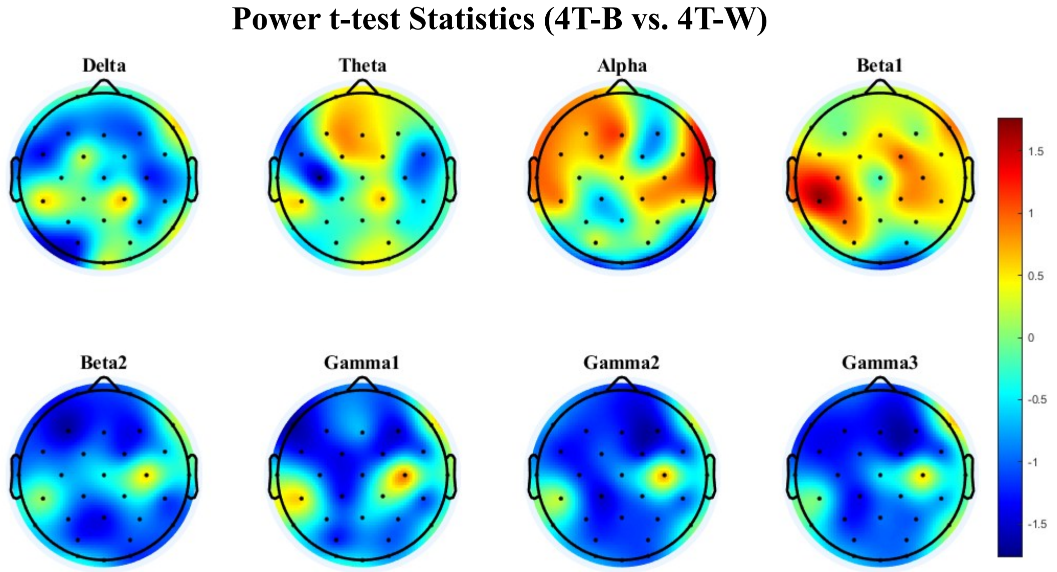

Figure S3: Topographical distributions of power t-test statistics (4T-B vs. 4T-W) across eight frequency bands. The color scale represents t-values, with warmer colors indicating higher power in the 4T-B condition and cooler colors indicating higher power in the 4T-W condition. No statistically significant differences were observed between conditions across any frequency band or electrode location.

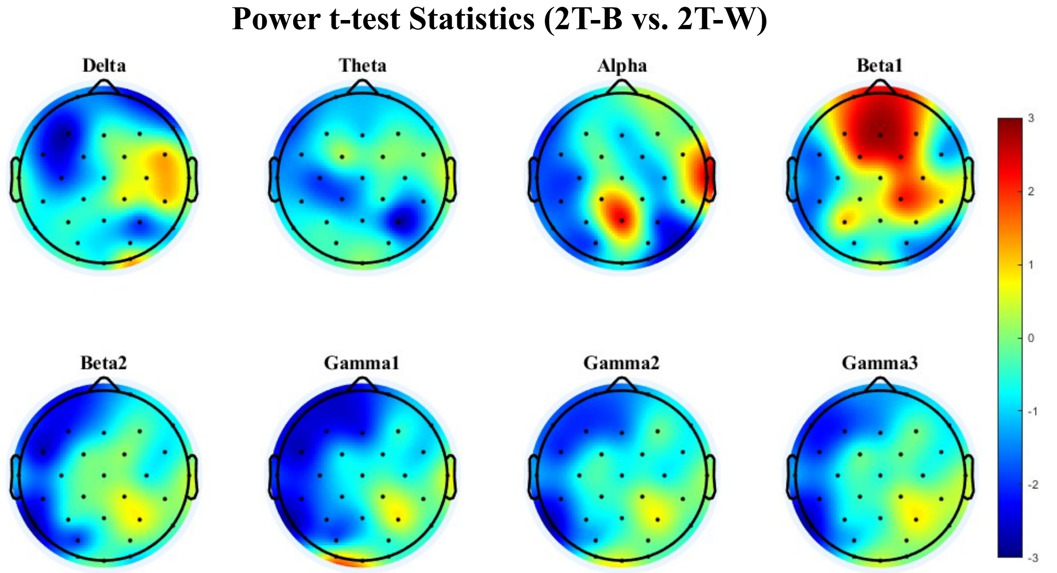

Figure S4: Topographical distributions of power t-test statistics (2T-B vs. 2T-W) across eight frequency bands.

6 PLV brain functional connectivity networks

Figures S5–S11 show the differences in the PLV networks in different frequency bands in the 4T-W and 4T-B conditions. The red and blue lines indicate significant increases and decreases, respectively. The differences were calculated using the Wilcoxon rank-sum test (FDR-corrected).

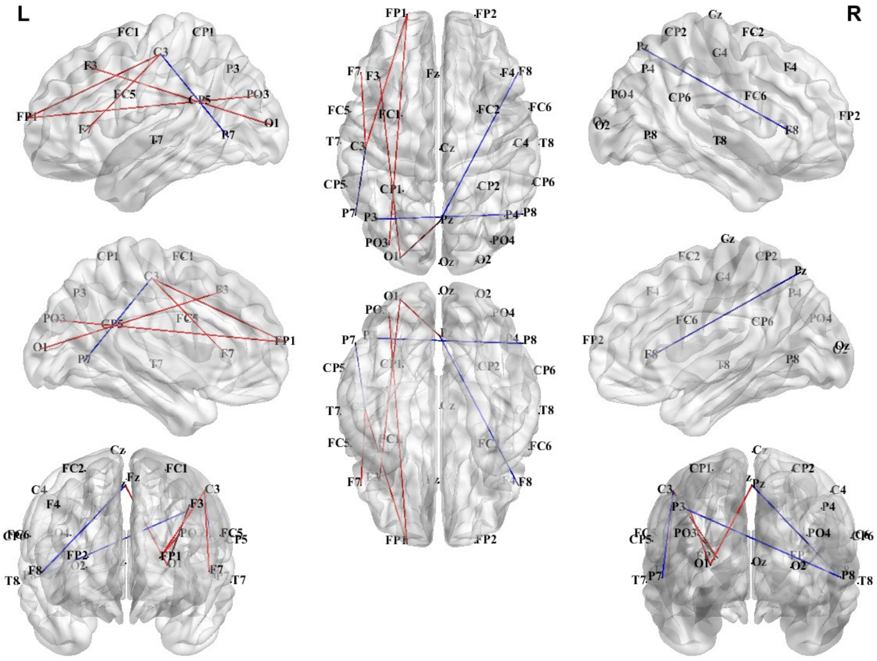

Figure S5: Differences in the brain functional networks in the delta band in the 4T-W and 4T-B conditions.

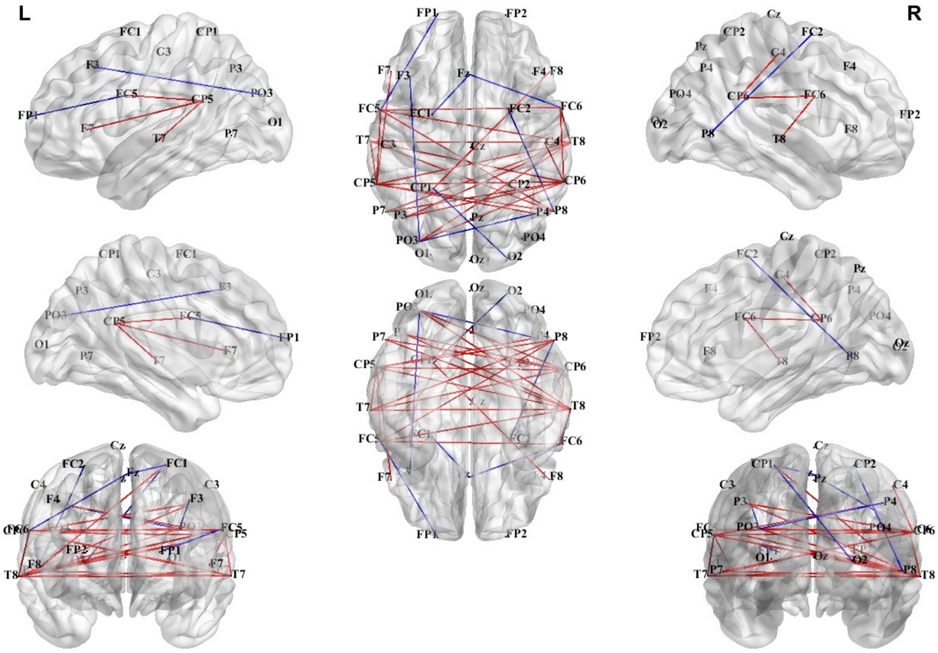

Figure S6: Differences in the brain functional networks in the alpha band in the 4T-W and 4T-B conditions.

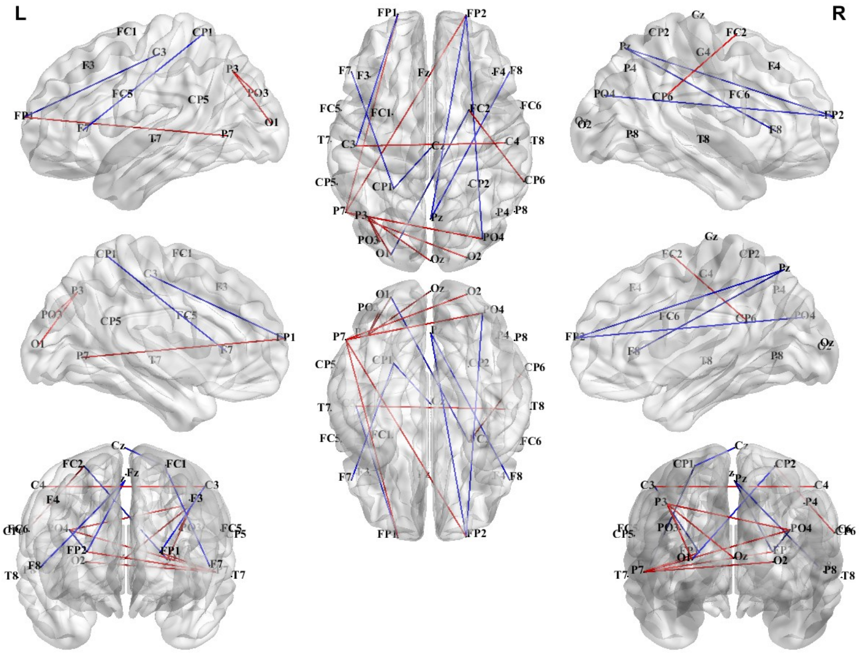

Figure S7: Differences in the brain functional networks in the beta1 band in the 4T-W and 4T-B conditions.

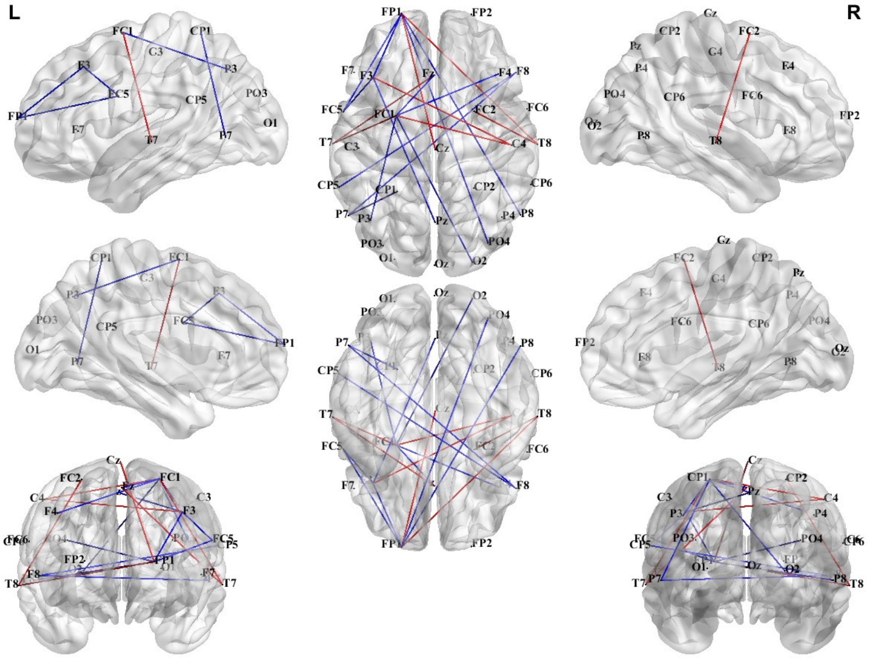

Figure S8: Differences in the brain functional networks in the beta2 band in the 4T-W and 4T-B conditions.

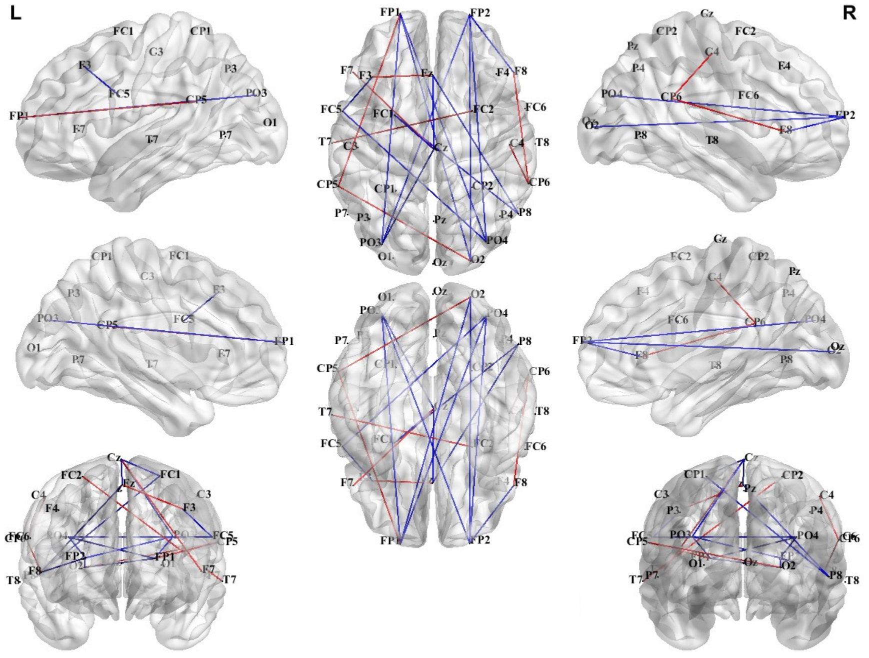

Figure S9: Differences in the brain functional networks in the gamma1 band in the 4T-W and 4T-B conditions.

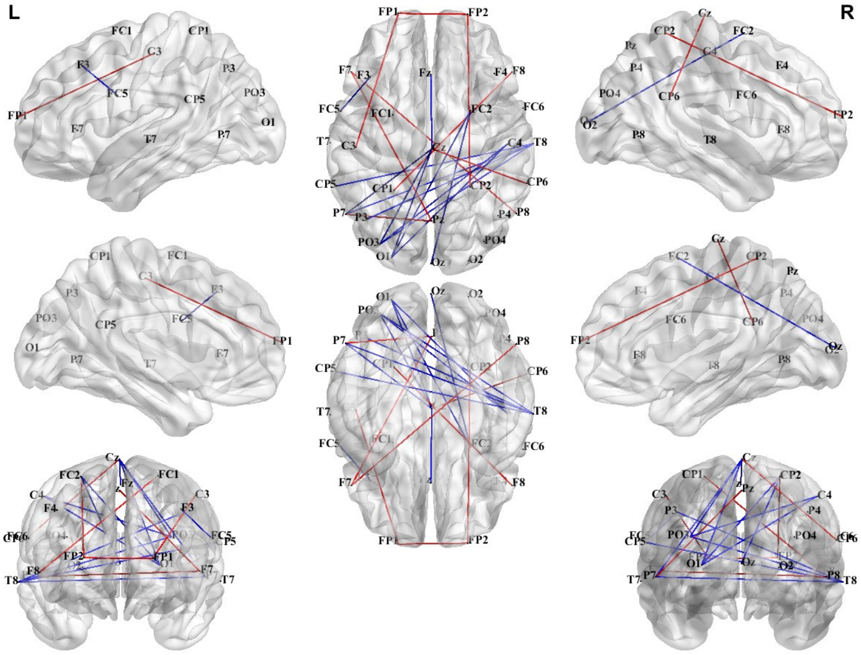

Figure S10: Differences in the brain functional networks in the gamma2 band in the 4T-W and 4T-B conditions.

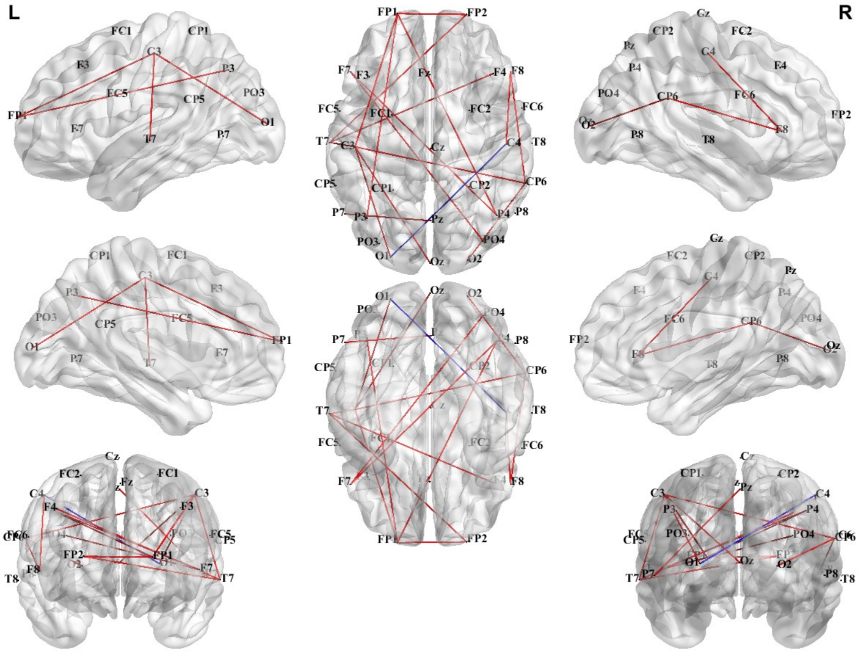

Figure S11: Differences in the brain functional networks in the gamma3 band in the 4T-W and 4T-B conditions.

7 Channel categorization

We employed a 32-channel EEG system based on the international 10–20 system. We divided all channels into six brain regions, including the frontal, central, left temporal, right temporal, parietal, and occipital regions, as shown in Appendix Figure S12.

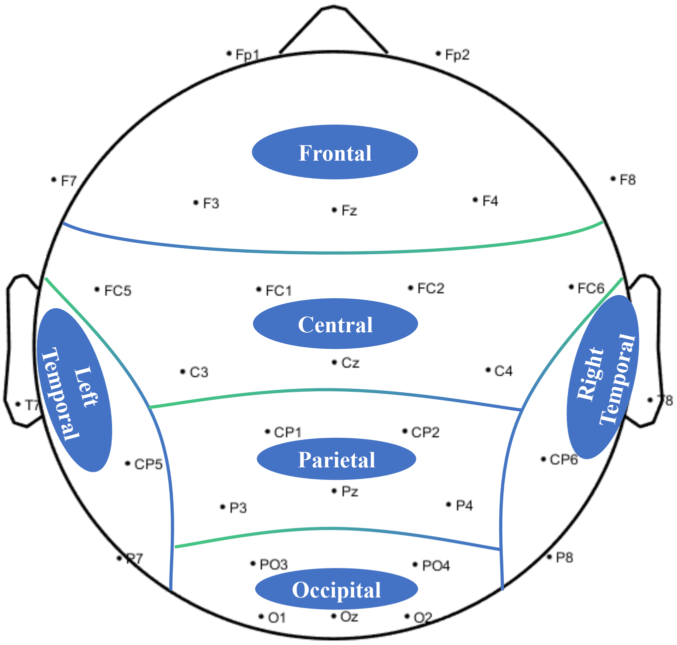

Figure S12: Brain topography and regions covered by electrodes.
